# Adipose mesenchymal stem cell-derived exosomes promote skin wound healing in diabetic mice by regulating epidermal autophagy

**DOI:** 10.1093/burnst/tkae001

**Published:** 2024-02-29

**Authors:** Haiyue Ren, Peng Su, Feng Zhao, Qiqi Zhang, Xing Huang, Cai He, Quan Wu, Zitong Wang, Jiajie Ma, Zhe Wang

**Affiliations:** Department of Pathology, Shengjing Hospital of China Medical University, 36 Sanhao Street, Heping District, Shenyang City 110004, Liaoning Province, China; Department of Pathology, Wuhan Hospital of Traditional Chinese and Western Medicine (Wuhan No.1 Hospital), Tongji Medical College, Huazhong University of Science and Technology, Wuhan, Hubei 430022, China; Medical Research Center, Shengjing Hospital of China Medical University, 36 Sanhao Street, Heping District, Shenyang City 110004, Liaoning Province, China; Department of Stem Cells and Regenerative Medicine, Shenyang Key Laboratory of Stem Cell and Regenerative Medicine, China Medical University, Shenyang 110013, Liaoning, China; Department of Pathology, Shengjing Hospital of China Medical University, 36 Sanhao Street, Heping District, Shenyang City 110004, Liaoning Province, China; Department of General Surgery, Shengjing Hospital of China Medical University, 36 Sanhao Street, Heping District, Shenyang City 110004, Liaoning Province, China; Department of Pathology, Shengjing Hospital of China Medical University, 36 Sanhao Street, Heping District, Shenyang City 110004, Liaoning Province, China; Department of Pathology, Shengjing Hospital of China Medical University, 36 Sanhao Street, Heping District, Shenyang City 110004, Liaoning Province, China; Department of Pathology, Shengjing Hospital of China Medical University, 36 Sanhao Street, Heping District, Shenyang City 110004, Liaoning Province, China; Department of Pathology, Shengjing Hospital of China Medical University, 36 Sanhao Street, Heping District, Shenyang City 110004, Liaoning Province, China; Department of Pathology, Shengjing Hospital of China Medical University, 36 Sanhao Street, Heping District, Shenyang City 110004, Liaoning Province, China

**Keywords:** Adipose-derived mesenchymal stem cells, Exosomes, Autophagy, Diabetes, Skin wound healing, Epidermal cells, Tissue repair, Regeneration

## Abstract

**Background:**

Adipose mesenchymal stem cell-derived exosomes (ADSC-Exos) have great potential in the field of tissue repair and regenerative medicine, particularly in cases of refractory diabetic wounds. Interestingly, autophagy plays a role in wound healing, and recent research has demonstrated that exosomes are closely associated with intracellular autophagy in biogenesis and molecular signaling mechanisms. Therefore, this study aimed to investigate whether ADSC-Exos promote the repair of diabetic wounds by regulating autophagy to provide a new method and theoretical basis for the treatment of diabetic wounds.

**Methods:**

Western blot analysis and autophagy double-labelled adenovirus were used to monitor changes in autophagy flow in human immortalized keratinocyte cell line (HaCaT) cells. ADSC-Exos were generated from ADSC supernatants via ultracentrifugation. The effectiveness of ADSC-Exos on HaCaT cells was assessed using a live-cell imaging system, cell counting kit-8 and cell scratch assays. The cells were treated with the autophagy inhibitor bafilomycin A1 to evaluate the effects of autophagy on cell function. The recovery of diabetic wounds after ADSC-Exo treatment was determined by calculating the healing rates and performing histological analysis. High-throughput transcriptome sequencing was used to analyze changes in mRNA expression after the treatment of HaCaT cells with ADSC-Exos.

**Results:**

ADSC-Exos activated autophagy in HaCaT cells, which was inhibited by high glucose levels, and potentiated their cellular functions. Moreover, ADSC-Exos in combination with the autophagy inhibitor bafilomycin A1 showed that autophagy defects further impaired the biological function of epidermal cells under high-glucose conditions and partially weakened the healing effect of ADSC-Exos. Using a diabetes wound model, we found that ADSC-Exos promoted skin wound healing in diabetic mice, as evidenced by increased epidermal autophagy and rapid re-epithelialization. Finally, sequencing results showed that increased expression of autophagy-related genes nicotinamide phosphoribosyltransferase (*NAMPT*), *CD46*, vesicle-associated membrane protein 7 (*VAMP7*), *VAMP3* and eukaryotic translation initiation factor 2 subunit alpha (*EIF2S1*) may contribute to the underlying mechanism of ADSC-Exo action.

**Conclusions:**

This study elucidated the molecular mechanism through which ADCS-Exos regulate autophagy in skin epithelial cells, thereby providing a new theoretical basis for the treatment and repair of skin epithelial damage by ADSC-Exos.

## Graphical Abstract

**Graphical Abstract:**
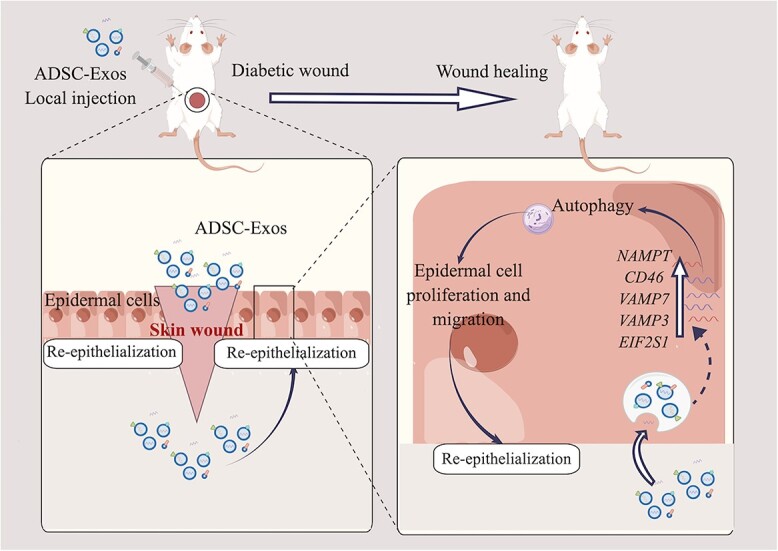

Highlights

- Cellular autophagy plays an important role in chronic wound healing in diabetes and a high-glucose environment reduces the level of cellular autophagy.
- ADSC-Exos promote diabetic wound healing by improving cell function damaged by a hyperglycemic environment by activating autophagy.
- The potential targets of ADSC-Exos activate autophagy and may be involved in the upregulation of the NAMPT, CD46, VAMP7, VAMP3 and EIF2S1 genes.

## Background

Diabetes has emerged as a rapidly growing health concern worldwide during the 21st century. The Global Diabetes Map (10^th^ Edition) published on the International Diabetes Federation website in 2021 indicates that the number of adults with diabetes reached 537 million (~1 in 10 adults) in 2021 and is expected to reach 784 million by 2045 [1]. In addition to its high prevalence, diabetes is associated with several co-morbidities. Chronic diabetic wounds, such as a diabetic foot ulcer (DFU), are the most common complications and the leading cause of disability and mortality among patients with diabetes [2,3]. Approximately 50–70% of all amputations have been reported to result from DFUs, and a leg is amputated every 30 s worldwide because of DFUs [4]. The mechanism of refractory diabetic wounds is complex. Although studies on chronic diabetic wounds are ongoing, strategies to effectively prevent and treat them are a significant problem.

The biological process of skin wound healing is complex and consists of three steps: inflammatory response, re-epithelialization and tissue remodelling [5,6]. Wound repair cells collaborate to complete the reconstruction of the skin barrier. Keratinocytes are the main components of epidermal cells. Wound re-epithelialization, an essential part of restoring skin barrier integrity, is an important process that is initiated early and continues throughout wound healing. It is largely dependent on the proliferation, migration and differentiation of epidermal cells [7]. Increased blood glucose levels, advanced glycation end products, reactive oxygen species (ROS) and proinflammatory cytokine aggregation in the diabetic wound microenvironment contribute to epidermal cell dysfunction, thereby delaying wound healing [8,9].

Autophagy is an important form of lysosomal metabolism in organisms and plays an essential role in regulating the normal physiological processes of cells [10]. Studies have indicated that autophagy, as a regulator of cellular homeostasis, is implicated in various stages of wound repair and plays a pivotal role in the orderly and expedited restoration of wounds [11–13]. A hyperglycemic environment results in skin cell dysfunction, including mitochondrial dysfunction, ROS accumulation, oxidative stress and inflammatory process overactivation, thereby leading to the development of refractory diabetic wounds [14]. Autophagy can remove damaged mitochondria through various pathways to prevent ROS accumulation, thereby reducing oxidative stress levels [15]. In addition, autophagy is conducive to the survival, migration and proliferation of cells required for tissue repair [16–18]. Overall, autophagy dysfunction significantly contributes to refractory diabetic wounds; however, its role in the wound healing process needs to be further elucidated. Targeting cellular autophagy represents a new approach for the treatment of diabetic refractory wounds.

Adipose mesenchymal stem cell-derived exosomes (ADSC-Exos) promote tissue repair and regeneration and are effective in reducing the immune response; moreover, they have a lower risk of tumour development than original stem cell therapies [19,20]. Therefore, ADSC-Exos exhibit great potential in tissue repair and regeneration. Although recent studies have confirmed that ADSC-Exos improve the cellular function of diabetic and non-diabetic wounds, the underlying mechanism remains unclear [21–24].

In this study, the effect of high glucose (HG) and ADSC-Exos on the regulation of epidermal cell function and autophagy was examined by establishing *ex vivo* and *in vivo* diabetic models. ADSC-Exo treatment promoted diabetic wound repair by restoring autophagy and function of epidermal cells inhibited by HG. To identify the key active components of ADSC-Exos in the specific regulation of cell autophagy, we performed transcriptome sequencing of the treated cells to screen for potential targets of ADSC-Exos involved in autophagy activation; this provides a new strategy for targeted gene therapy.

## Methods

### ADSC isolation and identification

All experimental procedures were approved by the Ethics Committee of Shengjing Hospital, China Medical University (No. 2023PS783K and No. 2023PS787K). Patients who had undergone abdominal surgery at the Department of General Surgery, Shengjing Hospital, China Medical University, were recruited to donate adipose tissue. Written informed consent was provided by all patients. ADSCs were isolated as described previously, i.e. they were obtained and co-cultured in mesenchymal stem cell medium (Yocon, Beijing, China) at 37°C under 5% CO_2_ to obtain stable ADSCs (3–6 passages) [25]. Flow cytometry (BD Biosciences, San Jose, CA, USA) was used to determine ADSC surface marker expression (CD45, CD73 and CD90). Antibodies were obtained from BD Biosciences. In addition, the multipotency of ADSCs was confirmed via alizarin red (Beyotime, Shanghai, China), oil red O (Beyotime) and alcian blue staining (Beyotime).

### Exosome isolation and identification

Supernatant was collected after culturing ADSCs in Dulbecco’s modified Eagle’s medium and nutrient mixture F-12 medium for 48 h. The supernatant was subjected to differential centrifugation at 300 × g for 5 min, 2000 × g for 15 min and 10 000 × g for 35 min. Then, the supernatant was filtered through a 0.22-μm filter (Millipore, MA, USA). Finally, the supernatant was transferred to a 100,000 molecular weight cut off (MWCO) ultrafiltration tube (Sartorius, Hamburg, Germany) and centrifuged at 3000 × g for 1.5 h. The supernatant was removed and the pellet was resuspended as ADSC-Exos with an appropriate amount of phosphate-buffered saline (PBS).

Exosome morphology was examined via transmission electron microscopy (TEM). Particle size distribution was determined via nanoparticle tracking analysis ( Particle Metrix, Germany). The presence of exosomal markers, including alix (rabbit IgG, Proteintech, Wuhan, China), CD9 (rabbit IgG, Proteintech), CD63 (rabbit IgG, Proteintech) and calnexin (rabbit IgG, Proteintech), was confirmed via western blot analysis as described below.

### Cell culture and treatment

A human immortalized keratinocyte cell line (HaCaT) was cultured in minimum essential medium (Procell, Wuhan, China) containing 10% fetal bovine serum (Procell), 1% 10 kU/ml penicillin and 10 mg/ml streptomycin (Procell) and incubated in a 5% CO_2_ incubator at 37°C and 95% humidity. HaCaT cells were treated with 35 mM glucose to generate a diabetic cell model. Mannitol (35 mM: 5.6 mM glucose + 29.4 mM mannitol) was used as an osmotic control for hyperglycemia. HaCaT cells were treated with 50–200 μg/ml ADSC-Exos. Bafilomycin A1 (BafA1, 10 nM; Sigma-Aldrich, MO, USA) was used to inhibit autophagy and added to HaCaT cells with or without ADSC-Exo treatment.

### Exosome labelling and tracking assay *in vitro* or *in vivo*

ADSC-Exos were stained with the exosomal red fluorescent marker PKH26 (Sigma-Aldrich) following the manufacturer’s instructions. PKH26-labelled exosomes (100 μg/ml) were co-cultured with HaCaT cells for 24 h, followed by fixation with 4% paraformaldehyde for 20 min. The cells were washed with PBS and stained with 4′,6-diamidino-2-phenylindole for 5 min. For *in vivo* studies, PKH26-labelled exosomes (2 μg/μl) were injected into the surrounding areas of the injured skin. Mice were sacrificed 5 h post injection. Subsequently, skin tissue cryosections were subjected to immunofluorescence staining. The primary antibody used was anti-pan-cytokeratin (AE1/AE3, sc-81 714; Santa Cruz Biotechnology, TX, USA). Finally, a confocal laser microscope (Zeiss, Oberkochen, Germany) was used to observe the entry of exosomes into the cells both *in vitro* and *in vivo*.

### Western blot analysis

Total protein was extracted and quantified using a total protein extraction kit (KeyGen Biotech, Nanjing, China) and BCA protein assay kit (EpiZyme, Shanghai, China), respectively, according to the manufacturer’s instructions. In total, proteins were separated by sodium dodecyl sulfate-polyacrylamide gel electrophoresis (SDS-PAGE) and transferred to Polyvinylidene fluoride (PVDF) membranes (Millipore, Billerica, MA, USA). The PVDF membranes were blocked at room temperature for 30 min using protein-free rapid blocking buffer (EpiZyme), followed by the addition of primary antibody and incubation overnight at 4°C with gentle shaking. The primary antibodies included anti-actin beta (ACTB, 1 : 3000; 205 361-AP, Proteintech), anti-microtubule associated protein 1 light chain 3 beta (MAP1LC3B/LC3B, 1 : 1000; ab48394, Abcam, Cambridge, UK) and anti-p62/sequestosome 1 (SQSTM1, 1 : 10,000; ab56416, Abcam). The membranes were washed three times with TBST for 5 min and incubated with horseradish peroxidase-conjugated secondary antibodies (1 : 10,000; SA00001–2, Proteintech) for 2 h at 25°C. Finally, color development and quantification were performed.

### Autophagy flux detection

HaCaT cells were seeded on crawlers and transfected with mRFP-GFP-LC3 adenovirus (multiplicity of infection = 100; Hanbio, HBAD-1007). The remaining steps were the as described in cell culture and treatment section.

### Cell proliferation assay

Cell proliferation capacity was evaluated using the cell counting kit-8 (CCK-8) assay (GK10001, GLPBIO, Montclair, USA). HaCaT cell suspensions (5 × 10^3^/well, 100 μl) were seeded into 96-well plates. Following incubation for the indicated time period, 10 μl of CCK-8 solution was added directly to each well and incubated for 2 h at 37°C. Absorbance was measured at 450 nm using a Gen5 plate reader (BioTek, Winooski, VT, USA). Cell confluence (percentage) was observed using IncuCyte ZOOM™ (Essen Bioscience, MI, USA); this instrument enables observation of cell growth, behavior and morphology. Moreover, its software can generate a proliferation growth curve of cells.

### Cell scratch assay

HaCaT cell migration ability was assessed using a scratch test. HaCaT cells were cultured in six-well plates for 48 h. The cells were subsequently scratched with a 200-μl pipette tip and photographed at 0 and 24 h. The relative scratch area size was calculated using ImageJ software (National Institutes of Health, Bethesda, MD, USA). The wound closure rate was calculated as follows: *W* (%) = [*S*_0_ − *S*_1_]/*S*_0_ × 100, where *W* is the wound closure rate and *S*_0_ and *S*_1_ are the wound areas at 0 and 24 h, respectively.

### Animal wound closure test

Animal testing was approved as described in ADSC isolation and identification section. A total of 30 normal adult Institute of Cancer Research (ICR) mice (male, 20–30 g) were obtained from HFK Bioscience Co. Ltd (Beijing, China). Diabetic mouse modelling was attained if fasting blood glucose measurements were ≥16.7 mmol/l on days 7 and 14 in the ICR mice after the administration of 120 mg/kg streptozotocin. Mice were anesthetized by intraperitoneal injection of sodium pentobarbital (0.5 mg/g) and a mouse skin defect model was generated. The mice were assigned to the following three groups: NDM + PBS group (non-diabetes mellitus mice injected with 100 μl of PBS after skin wound formation), DM + PBS group (diabetes mellitus mice injected with 100 μl PBS after skin wound formation) and DM + ADSC-Exos group (diabetes mellitus mice injected with 200 μg of ADSC-Exos dissolved in 100 μl of PBS after skin wound formation). The wounds were photographed on days 0, 3, 5, 7, 10 and 14, and wound area was calculated using ImageJ software. The percentage of wound closure area was calculated as follows: *P* (%) = [*A*_0_ − *A*_1_]/*A*_0_ × 100, where *P* is the percentage of wound closure and *A*_0_ and *A*_1_ are the initial wound area and residual wound area, respectively.

### Hematoxylin and eosin (H&E) staining

On days 7 and 14 of the animal study, skin wound recovery was assessed by performing H&E staining as described in a previous study [26].

### Immunohistochemical (IHC) staining

Paraffin sections were incubated at 60°C for 120 min. Dewaxing and hydration were performed using xylene, distilled water and decreasing concentrations of ethanol. For antigen repair, 10 mM sodium citrate repair solution was applied (95°C, 20 min). The sections were subsequently blocked with 5% bovine serum albumin (25°C, 30 min) and incubated with anti-LC3 (1:100, Proteintech) and anti-Beclin-1 (1100, Proteintech) antibodies sequentially overnight at 4°C. Subsequently, secondary antibodies were added dropwise and incubated for 60 min at 25°C. Diaminobenzidine was used for color development. Finally, the sections were observed under a light microscope and positive optical density was determined using ImageJ software.

### RNA sequencing

HaCaT cells were incubated with HG (35 mM) with or without ADSC-Exos (100 μg/ml) for 48 h (n = 3). Total RNA was isolated using an RNAiso Plus kit (Vazyme Biotech Co., Ltd, Nanjing, China) and mRNA was enriched using magnetic beads with oligo (dT). Following fragmentation, the mRNA was converted into individual cDNA libraries. The libraries were qualified and subsequently sequenced using Illumina NovaSeq 6000. A *p* value ≤ 0.05 and |log2foldchange| ≥ 0.0 were set as the threshold for significant differences.

### Quantitative reverse transcription polymerase chain reaction

Total RNA was isolated using an RNAiso Plus kit (Vazyme Biotech Co., Ltd). The RNA was reverse transcribed into cDNA using HiScript II qRT SuperMix kit for qPCR (Vazyme Biotech Co., Ltd). The SYBR Green PCR kit (Vazyme Biotech Co., Ltd) was used to perform quantitative reverse transcription polymerase chain reaction (qRT-PCR). The 2^−ΔΔCT^ method was used to quantify mRNA expression, with ACTB as a reference. The primer sequences are listed in Table 1.

**Table 1:** Sequences of primers used for qRT-PCR

**Target gene**	**Forward primer 5′–3′**	**Reverse primer 3′–5′**
*NAMPT*	GCAGAAGCCGAGTTCAACAT	CTTTGCTTGTGTTGGGTGGA
*CD46*	GAAAGCAGATGGTGGAGCTG	GGCAAACCAGGTTGTGGAAT
*VAMP7*	TAGGGCAATCGTGTCGCTAA	CCGTATTCGGTTTGGGCTTT
*VAMP3*	AGCTGGCAGTGTTAGGACAT	AGTGGGTTACATGGGTCTGG
*EIF2S1*	AGGCCTTTCTGTCCTCAGTC	TCTCCATCCACTTCGGCATT
*ACTB*	CATGTACGTTGCTATCCAGGC	CTCCTTAATGTCACGCACGAT

### Statistical analysis

All results were analyzed using Prism 9 software (GraphPad Software V9.0, CA, USA). Each experiment was performed more than three times. The data are expressed as mean ± standard deviation. Statistical differences between two groups were compared using t-tests, and those among more than two groups were compared using a one-way analysis of variance (ANOVA). Two-way ANOVA with multiple comparisons was used to compare significant differences for both time and treatment factors. Tukey’s *post hoc* test was performed following ANOVA. A *p* value < 0.05 was considered statistically significant.

## Results

### HG inhibits epidermal cell autophagy

**Figure 1:**
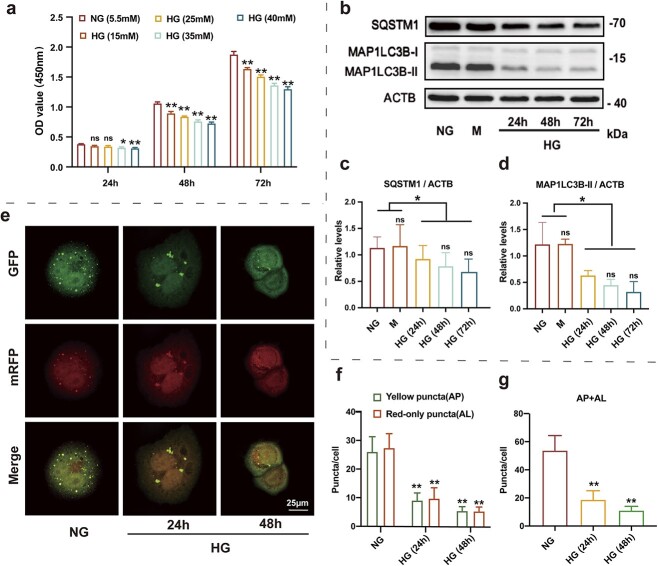
High glucose (HG) inhibits autophagy in epidermal cells. (**a**) CCK-8 assay was used to detect the effect of HG on cell proliferation. (**b**) Western blot analysis of SQSTM1, MAP1LC3B-I and MAP1LC3B-II expression levels in HaCaT cells treated with normal glucose (NG, 5.6 mM glucose) or mannitol (M, 35 mM: 5.6 mM glucose + 29.4 mM mannitol) for 72 h or HG (35 mM glucose) for 24, 48 and 72 h. (**c**, **d**) Quantification of MAP1LC3B-II/ACTB ratios and SQSTM1 protein expression levels (n = 3 independent experiments). (**e**) HaCaT cells were infected with adenovirus harboring tandem fluorescent mRFP–GFP–MAP1LC3B for 24 h, followed by treatment with NG (5.6 mM) or HG (35 mM) for 24 or 48 h. Representative images of HaCaT cells expressing mRFP–GFP–MAP1LC3B are shown, scale bar: 25 μm. (**f**, **g**) Semi-quantitative analysis of autophagosomes (AP, yellow puncta in merged images) and autolysosomes (AL, red-only puncta in merged images) in HaCaT cells (n = 3 independent experiments). Data are presented as mean ± SD. ^*^p < 0.05, ^**^p < 0.01 *vs* NG; ^#^*p* < 0.05 *vs* HG (24 h). *ns* not significant, *OD* optical density, *CCK-8* cell counting kit-8, *SQSTM1* sequestosome 1, *MAP1LC3B* microtubule associated protein 1 light chain 3 beta, *ACTB* actin beta, *HaCaT* human immortalized keratinocyte cell line

To simulate a HG environment similar to diabetes, HaCaT cells were studied in the presence of HG (15, 25, 35 and 40 mM) for 24, 48 and 72 h, and cell viability was determined using the CCK-8 assay (Figure 1a). Optical density values of other groups were lower than that of the normal glucose (NG; 5.5 mM) group. Glucose inhibited cell proliferation in a concentration-dependent manner. In accordance with a previous study we established a diabetic cell model using 35 mM glucose [27].

HaCaT cells were treated with HG (35 mM) for 24, 48 and 72 h as well as with NG (5.5 mM) and 35 mM mannitol as controls, the latter for osmolarity. Changes in autophagy in these cells were observed under HG conditions. Western blot analysis revealed that SQSTM1 expression levels and MAP1LC3B-II/ACTB ratios were significantly reduced following hyperglycemic treatment, whereas hyperosmotic treatment had no effect (Figure 1b–d).

At present, commonly used autophagy observation and monitoring strategies can not only detect the cleavage of MAPLC3B via western blot analysis but can also trace the formation and degradation of autophagosomes via fluorescence microscopy using fusion proteins, such as mRFP–GFP–MAPLC3B. Therefore, we assessed the autophagy flux of HaCaT cells under HG following transfection with mRFP–GFP–MAPLC3B autophagy double-label adenovirus. The results indicated that autophagosomes (yellow spots) and autolysosomes (red spots) were significantly down-regulated in HaCaT cells following HG stimulation (*p* < 0.05, Figure 1e–g). This indicates that HG (35 mM) can significantly impair autophagic flux in HaCaT cells.

### ADSC and ADSC-Exo isolation and identification

**Figure 2:**
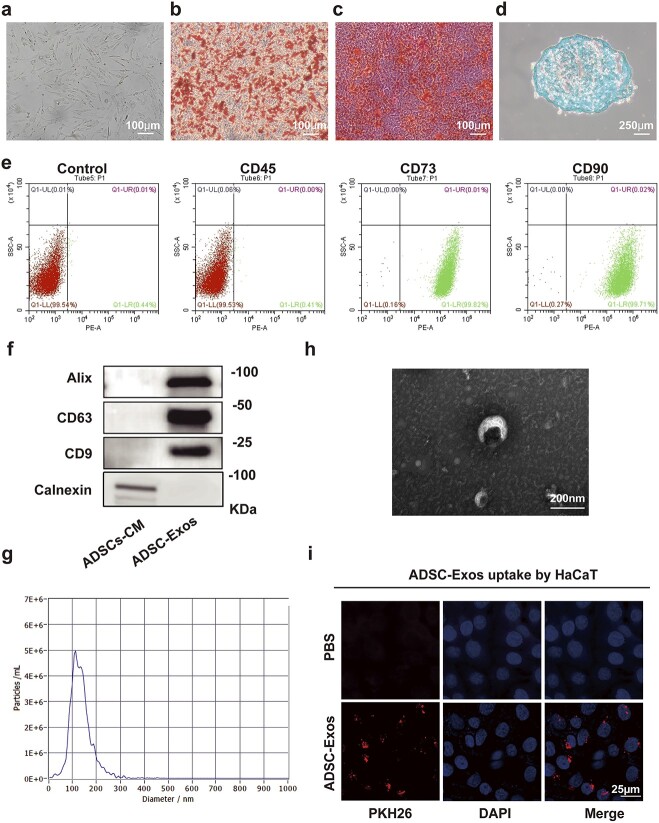
Identification of adipose mesenchymal stem cells (ADSCs) and adipose mesenchymal stem cell-derived exosomes (ADSC-Exos). Representative images of: (**a**) normal cultured ADSCs under a microscope, scale bar: 100 μm; (**b**) ADSCs after osteogenic differentiation, scale bar: 100 μm; (**c**) ADSCs after adipogenic differentiation, scale bar: 100 μm; and (**d**) ADSCs after chondrogenic differentiation, scale bar: 250 μm. (**e**) Flow cytometry analysis of ADSC surface markers: CD45, CD73, CD90. (**f**) Western blot analysis of ADSC-Exo markers: CD9, CD63,alix, and calnexin. (**g**) Nanoparticle tracking analysis (NTA) of isolated ADSC-Exos. (**h**) Transmission electron microscopy image of ADSC-Exo morphology, scale bar: 200 nm. (**i**) Fluorescent microscopy analysis of PKH26-labelled ADSC-Exo uptake by HaCaT cells, scale bar: 25 μm. *HaCaT* human immortalized keratinocyte cell line, *DAPI* 4′,6-diamidino-2-phenylindole, *PBS* phosphate-buffered saline

ADSCs showed a typical spindle-formed, fibroblast-like structure under a light microscope (Figure 2a). Alizarin red (Figure 2b), oil red O (Figure 2c) and alcian blue (Figure 2d) staining confirmed that ADSCs could differentiate into osteoblasts, adipocytes and chondrocytes. Flow cytometry analysis revealed that CD73 and CD90 (mesenchymal stem cells specific biomarkers) were highly expressed in ADSCs, whereas CD45 (haematopoietic cell marker) was not expressed (Figure 2e). These results indicated that ADSCs were successfully isolated.

Exosomes were isolated from ADSC supernatant via ultracentrifugation. Western blot analysis revealed that ADSC-Exos were enriched in exosomal markers, such as alix, CD63 and CD9, compared with ADSC-conditioned medium, whereas calnexin was absent (Figure 2f). Nanoparticle tracking analysis was used to calculate the particle size of ADSC-Exos from the trajectory of Brownian motion. The particle size of most ADSC-Exos was found to be primarily distributed around 110 nm (Figure 2g). TEM observations revealed that ADSC-Exos had a characteristic cup-shape and double-capsule membrane ultrastructure (Figure 2h). These results indicated that ADSC-Exos were successfully isolated from the conditioned medium of ADSCs.

### ADSC-Exos induce proliferation, migration and autophagy of HG-treated epidermal cells

To further elucidate the effect of ADSC-Exos on epidermal cells, we first verified whether ADSC-Exos were able to enter HaCaT cells and exert biological effects. The results indicated that the red fluorescent dye (PKH26)-labelled ADSC-Exos, after incubation with HaCaT cells for 24 h, were endocytosed into the cytoplasm and accumulated around the nucleus, which was labelled with DAPI (Figure 2i).

**Figure 3:**
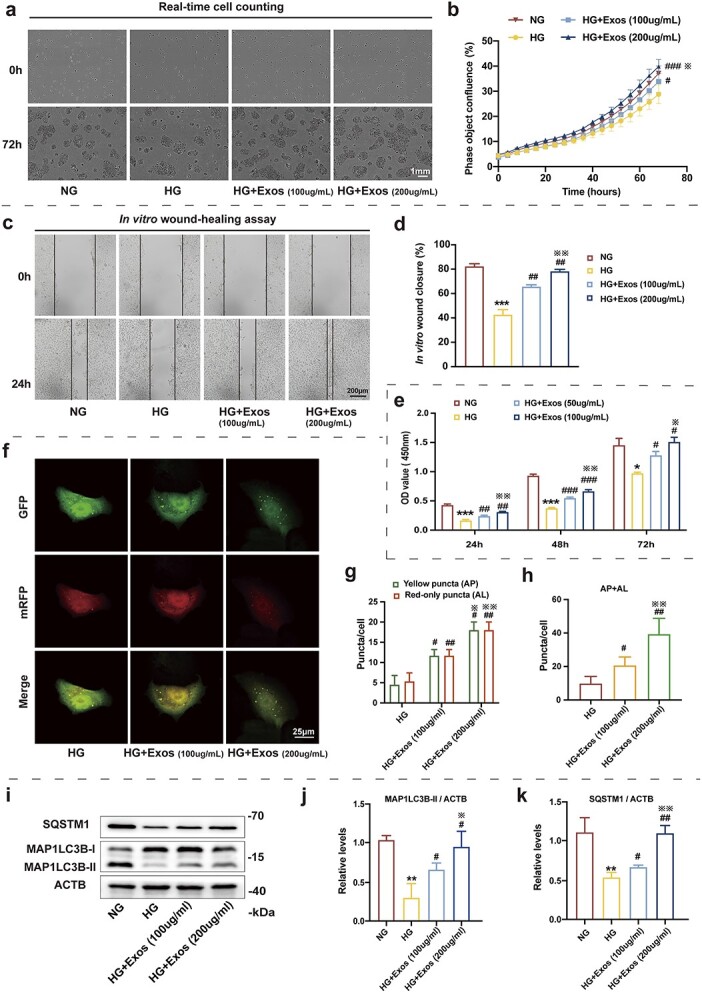
Adipose mesenchymal stem cell-derived exosomes (ADSC-Exos) induce the proliferation, migration and autophagy of epidermal cells treated with high glucose (HG). (**a**) Representative images of cell proliferation detected by Incucyte Zoom live cell imaging, scale bar: 1 mm. (**b**) Incucyte Zoom live cell imaging showing the phase object confluence of HaCaT cells under different conditions. (**c**, **d**) Representative images of *in vitro* wound-healing assays with HaCaT cells treated with ADSC-Exos under HG. Images were captured at 0 and 24 h after the scratch. Semi-quantitative analysis of wound-healing assays (n = 3 independent experiments). (**e**) CCK-8 assay was used to detect the effect of ADSC-Exos under HG on cell proliferation. (**f–h**) HaCaT cells were infected with adenovirus harbouring tandem fluorescent mRFP–GFP–MAP1LC3B for 24 h, followed by treatment with HG (35 mM), HG + ADSC-Exos (100 μg/ml) or HG + ADSC-Exos (200 μg/ml) for an additional 48 h. Representative images of HaCaT cells expressing mRFP–GFP–MAP1LC3B are shown, scale bar: 25 μm. Semi-quantitative analysis of autophagosomes (AP, yellow puncta in merged images) and autolysosomes (AL, red-only puncta in merged images) in HaCaT cells are shown (n = 3 independent experiments). (**i**) Western blot analysis of SQSTM1, MAP1LC3B-I and MAP1LC3B-II expression levels in HaCaT cells treated with NG (5.6 mM), HG (35 mM), HG + ADSC-Exos (100 μg/ml) or HG + ADSC-Exos (200 μg/ml) for 48 h. (**j**, **k**) Quantification of MAP1LC3B-II/ACTB ratios and SQSTM1 protein expression levels are shown (n = 3 independent experiments). Data are presented as mean ± SD. ^*^p < 0.05, ^**^p < 0.01, ^***^p < 0.001 *vs* NG; ^#^*p* < 0.05, ^##^*p* < 0.01, ^###^*p* < 0.001 *vs* HG; ^⋇^*p *< 0.05, ^⋇^^⋇^*p* < 0.01 *vs *HG + Exos (100 μg/ml)*. HaCaT* human immortalized keratinocyte cell line, *CCK-8* cell counting kit-8, *SQSTM1* sequestosome 1, *MAP1LC3B* microtubule associated protein 1 light chain 3 beta, *ACTB* actin beta, *OD* optical density

Subsequently, a live cell imaging system (Figure 3a and b), an *in vitro* wound healing model (Figure 3c and d) and CCK-8 assay (Figure 3e) were used to determine the regulatory effect of ADSC-Exo co-incubation on HaCaT cell proliferation and migration in a HG (35 mM) environment. HG resulted in a significant reduction in cell growth and migration, whereas ADSC-Exos treatment restored these functions in a concentration-dependent manner.

We further evaluated the beneficial effects of ADSC-Exos on HaCaT cell autophagy. ADSC-Exos promoted autophagosome and autolysosome production in HaCaT cells, thereby suggesting that ADSC-Exos improved autophagic flux in a HG environment (Figure 3f–h). Moreover, we found that the reduction of the relative SQSTM1 expression level and MAP1LC3B-II/ACTB ratio due to HG levels was significantly up-regulated by ADSC-Exo treatment (Figure 3i–k). In addition, we treated HaCaT cells with an autophagy activator (mammalian target of rapamycin complex 1 (mTORC1) inhibitor) as a positive control. The mTORC1 inhibitor markedly elevated the decrease in the MAP1LC3B-II/ACTB ratio induced by elevated glucose concentration, which was consistent with the effect of ADSC-Exos (Figure S1a, see online supplementary material).

### Inhibition of autophagy adversely affects the positive effect of ADSC-Exos on HaCaT cells under HG conditions

**Figure 4:**
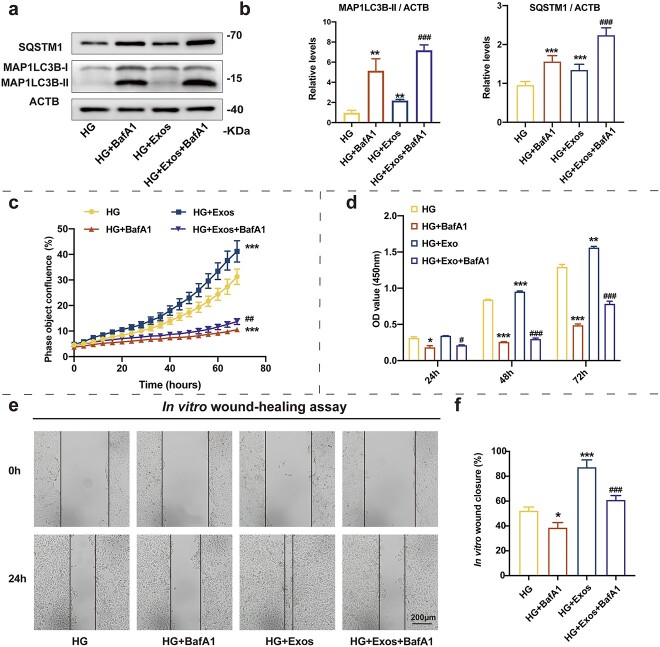
Effect of adipose mesenchymal stem cell-derived exosomes (ADSC-Exos) on enhancing HaCaT cell function in high glucose (HG) was blocked by inhibiting autophagy. (**a**,**b**) Western blot analysis of SQSTM1, MAP1LC3B-I and MAP1LC3B-II expression levels in HaCaT cells treated with HG (35 mM), HG + BafA1 (10 nM), HG + ADSC-Exos (200 μg/ml) or HG + ADSC-Exos + BafA1 for 48 h. Quantification of MAP1LC3B-II/ACTB ratios and SQSTM1 protein expression levels are shown (n = 3 independent experiments). (**c**) Incucyte Zoom live cell imaging showing the phase object confluence of HaCaT cells under different conditions. (**d**) CCK-8 assay showing cell proliferation under different conditions. (**e**, **f**) Representative images of *in vitro* wound-healing assays in HaCaT cells treated under different conditions. Images were captured at 0 and 24 h after the scratch. Semi-quantitative analysis of wound-healing assays (n = 3 independent experiments). Data are presented as mean ± SD. ^*^p < 0.05, ^*^^*^p < 0.01, ^*^^*^^*^p < 0.001* vs* HG; ^#^*p* < 0.05, ^##^*p* < 0.01, ^###^*p* < 0.001 *vs* HG + Exos. *HaCaT* human immortalized keratinocyte cell line, *CCK-8* cell counting kit-8, *SQSTM1* sequestosome 1, *MAP1LC3B* microtubule associated protein 1 light chain 3 beta, *ACTB* actin beta, *BafA1* bafilomycin A1

BafA1 is a commonly used autophagy inhibitor that prevents the fusion of autophagy vesicles and lysosomes by inhibiting lysosomal acidification. This blocks autophagy degradation and results in the accumulation of autophagy substrates. In the present study, BafA1 was used to inhibit the autophagy of HaCaT cells, and the role of ADCS-Exos on the function of HaCaT cells was examined. MAPLC3B II and SQSTM1 accumulation in the cells indicated that BafA1 significantly reduced autophagic flux (Figure 4a and b). The live cell imaging system and CCK-8 proliferation assay revealed that ADSC-Exos had a reduced ability to promote HaCaT cell proliferation because of autophagy defects (Figure 4c and d). An effect of the autophagy defect on HaCaT cell migration was observed and ADSC-Exos were unable to restore HaCaT cell trauma repair ability or migration because of defective HaCaT cell autophagy (Figure 4e and f). Based on these results, we hypothesized that ADSC-Exos may enhance HaCaT cell function by regulating the autophagy of epidermal cells.

### ADSC-Exos enhance wound healing in diabetic mice

To determine the effect of ADSC-Exos on chronic diabetic wound healing *in vivo*, 30 ICR mice were procured. Diabetes was induced in 21 of them. Two mice died after intraperitoneal injection with streptozotocin solution, whereas the remaining 19 mice exhibited a random blood glucose level of ≥16.7 mmol/l. The mice in the model group showed signs of weight loss, polyuria, polydipsia and polyphagia compared with the healthy control group. The success rate of the model was >90%.

**Figure 5:**
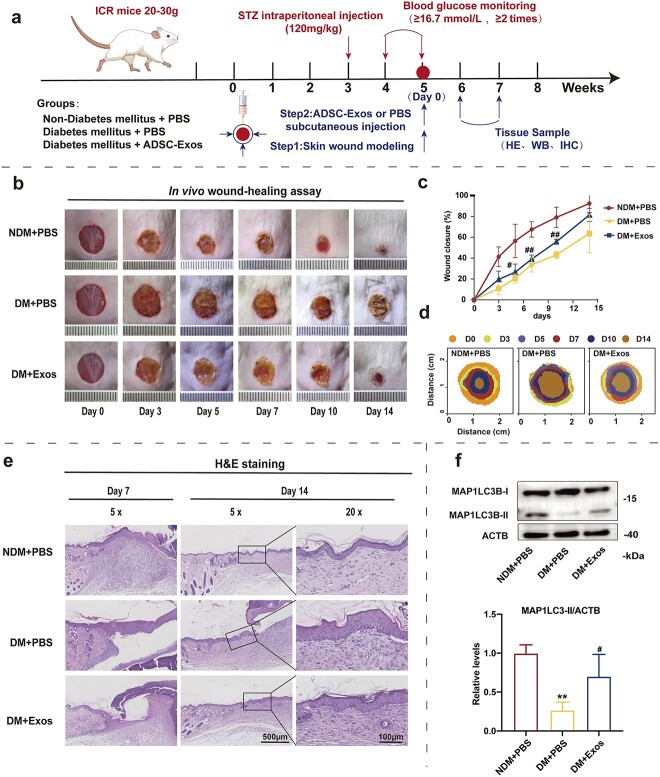
Observation of wound healing quality and rate following operation. (**a**) Generating a full-thickness skin defect model and treatment protocol. (**b**) Representative images of wounds treated with PBS or ADSC-Exos. (**c**) Wound closure percentage statistics and (**d**) wound traces. (**e**) H&E staining of wound sections on days 7 and 14 following operation. (**f**) Western blot analysis of MAP1LC3B-I and MAP1LC3B-II expression levels in wound tissue on day 7 post operation. Quantification of the MAP1LC3B-II/ACTB ratios is shown. Data are presented as mean ± SD. ^**^p < 0.01* vs* NDM + PBS. ^#^*p* < 0.05, ^##^*p* < 0.01 *vs* DM + PBS. *PBS* phosphate-buffered saline, *ADSC-Exos* adipose mesenchymal stem cell-derived exosomes, *H&E * hematoxylin and eosin, *MAP1LC3B* microtubule associated protein 1 light chain 3 beta, *ACTB* actin beta, *NDM* non-diabetes mellitus mice, *DM* diabetes mellitus mice, *STZ* streptozotocin, *ICR* Institute of Cancer Research, *WB* western blot, *IHC* immunohistochemistry

Next, a full-thickness skin defect was created on the back of the mice; the treatment protocol is presented in Figure 5a. Specific images and dimensions among other characteristics of the trauma are shown in Figure 5b–d. Although the wounds became progressively smaller in all groups under different treatment modalities, the healthy control (NDM + PBS) group exhibited a significantly greater wound healing rate than the DM + PBS and DM + ADSC-Exos groups. Moreover, the DM + Exo group exhibited rapid wound closure compared with the DM + PBS group, with smaller wound areas observed on days 3, 5, 7, 10 and 14. H&E staining was used to evaluate histological changes in each group. The DM + PBS group exhibited a lower wound healing rate than the NDM + PBS group, with dermal epidermis separation and epidermal cells in a disordered arrangement on days 7 and 21. ADSC-Exo treatment (DM + ADSC-Exos group) ameliorated the changes in the DM + PBS group, with a more complete wound epithelium and greater skin appendage formation (such as hair follicles) (Figure 5e). This suggests that ADSC-Exos facilitate skin wound healing in diabetic mice and epidermal regeneration. Using an *in vivo* tracking assay, we observed striking co-localization of exosomes (PKH26, red) and epidermal cells (pan-cytokeratin, green) at locations adjacent to the injury sites, verifying the absorption of exosomes by epidermal cells (Figure S1b, see online supplementary material).

**Figure 6:**
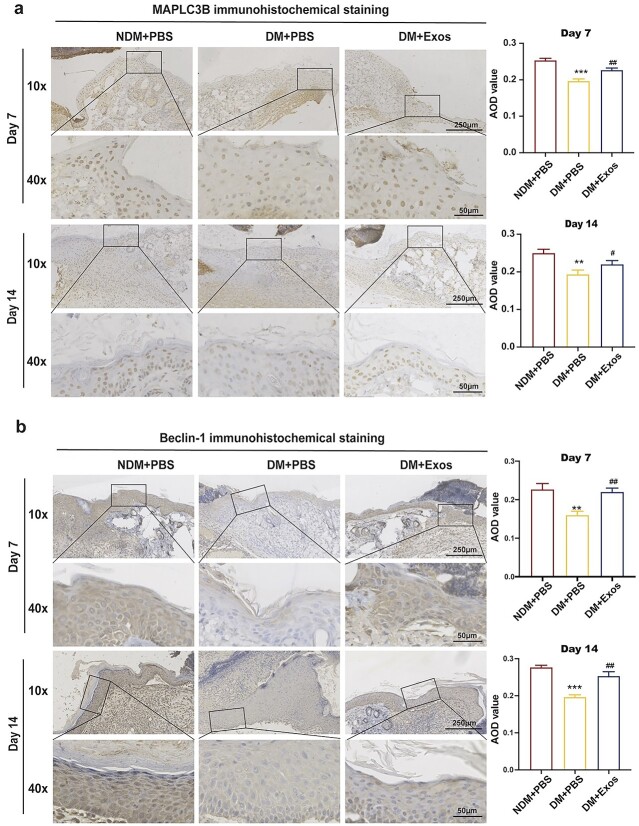
Histological analysis of wound healing in different groups. (**a**) MAP1LC3B and Beclin-1 expression in wound tissue was observed on days 7 and 14 following operation. (**b**) Semi-quantitative analysis of MAP1LC3B and Beclin-1 in wound tissue observed on days 7 and 14 following operation. Data are presented as mean ± SD. ^**^p < 0.01, ^***^p < 0.001 *vs* NDM + PBS; ^#^*p* < 0.05, ^##^*p* < 0.01 *vs* DM + PBS. *MAP1LC3B* microtubule-associated protein 1 light chain 3 beta, *NDM* non-diabetes mellitus mice, *DM* diabetes mellitus mice, *PBS* phosphate-buffered saline

**Figure 7:**
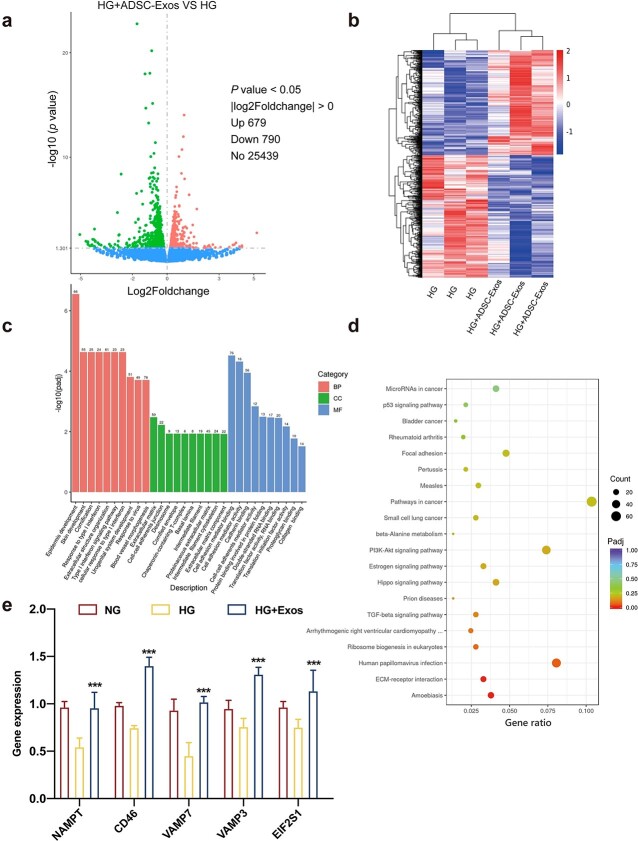
High-throughput sequencing analysis of differentially expressed genes in HaCaT cells treated with high glucose (HG) or HG + ADSC-Exos. (**a**) Volcano plot of differentially expressed mRNAs depicting up-regulated and down-regulated genes. (**b**) Heat map showing differentially expressed genes in HaCaT cells treated with HG or HG + ADSC-Exos. (**c**) Results of GO analysis. (**d**) Results of KEGG analysis. (**e**) qRT-PCR analysis of the mRNA expression levels of differentially up-regulated ARGs in HaCaT cells. Data are presented as mean ± SD. ^***^p < 0.001 *vs* HG. *HaCaT* human immortalized keratinocyte cell lines, *ADSC-Exos* adipose mesenchymal stem cell-derived exosomes, *GO* gene ontology, *KEGG* Kyoto encyclopedia of genes and genomes

### ADSC-Exos activate autophagy to promote skin wound repair in diabetic mice

To further examine the effect of autophagy on the promotion of skin wound repair in diabetic mice by ADSC-Exos, western blot analysis was performed on skin tissues on day 7 after the establishment of the skin wound model. The results indicated that the MAP1LC3B-II/ACTB ratio was decreased in the DM + PBS group compared with that in the NDM + PBS group, whereas the MAP1LC3B-II/ACTB ratio was increased in the DM + Exos group compared with that in the DM + PBS group (Figure 5f).

Moreover, we conducted immunohistochemical staining on days 7 and 14 following the establishment of the skin wound model and found that the changes in MAPLC3B expression were consistent with those observed in western blotting (Figure 6a).

We also performed IHC staining for Beclin-1, another autophagy marker, to observe the role of autophagy. Changes in Beclin-1 expression were consistent with those observed in MAPLC3B expression. On days 7 and 14, Beclin-1 expression in the DM + PBS group was decreased compared with that in the NDM + PBS group, whereas Beclin-1 expression in the DM + Exos group was increased compared with that in the DM + PBS group (Figure 6b). Therefore, we concluded that autophagy levels were decreased in the skin tissue of diabetic mice and that ADSC-Exos activated autophagy to accelerate wound repair.

### Potential mechanism of ADSC-Exos in activating autophagy to promote wound repair in diabetic mice

High-throughput transcriptome sequencing was used to analyze changes in mRNA expression within 48 h following ADSC-Exo treatment of HaCaT cells to identify a potential mechanism through which ADSC-Exos activate autophagy to promote diabetic wound repair. We designated a *p* value of ≤0.05 and a |log2foldchange| value of ≥0.00 to determine whether there was significant differential expression of a particular gene. In total, 679 up-regulated and 790 down-regulated mRNAs were identified as shown in the volcano plot and heat map (Figure 7a and b). Gene ontology (GO) enrichment (Figure 7c) and Kyoto encyclopedia of genes and genomes (KEGG) pathway (Figure 7d) analyses were used to determine the association of differentially expressed genes (DEGs) with specific biological processes or pathways. The results indicated that DEGs were involved in biological processes related to the epidermis and skin development. Most DEGs were associated with the extracellular matrix and cell–cell adherens junctions. In addition, the role of these molecules involved cell adhesion molecule binding, cell adhesion mediator activity and cadherin binding. Moreover, KEGG pathways, including amoebiasis, extracellular matrix–receptor interaction and human papillomavirus infection, were enriched in ADSC-Exo-treated HaCaT cells. The KEGG pathway that most interested us was autophagy; thus, we selected several differentially expressed autophagy-related genes (ARGs) present in the pathway for preliminary studies. qRT-PCR revealed that among these genes, *NAMPT* (nicotinamide phosphoribosyltransferase), *CD46*, *VAMP7* (vesicle-associated membrane protein 7), *VAMP3* and *EIF2S1* (eukaryotic translation initiation factor 2 subunit alpha) were expressed at levels consistent with the sequence analysis results. Interestingly, these ARGs were significantly down-regulated in the HG group compared with those in the NG group (Figure 7e).

## Discussion

Our results indicated that a high-sugar environment inhibits autophagy in epidermal cells and impairs certain biological functions, such as proliferation and migration. ADSC-Exos can activate autophagy in epidermal cells and promote their proliferation and migration. Moreover, we found that defects in autophagy further impair the biological function of epidermal cells under HG conditions and partially offset the healing effect of ADSC-Exos. In addition, the animal model revealed that ADSC-Exos regulate autophagy and enhance epidermal epithelialization, which in turn promotes trauma regeneration. Overall, our results demonstrate the influence of ADSC-Exos on the regulation of cellular autophagy in diabetic wounds and represent a new approach for wound repair.

Autophagy, a cellular quality control mechanism, is a key intracellular catabolic process for the elimination of damaged proteins and organelles. In recent years, reduced autophagy levels have been reported to be strongly associated with the development of diabetes [28–32]. The results of a clinical trial revealed that MAPLC3A/B, an autophagy marker, was highly expressed in patients with diabetes and that mitochondrial DNA was replicated at high levels [33]. However, the autophagy monitoring method used in the present study could not accurately detect MAPLC3A/B cleavage; thus, we could not determine autophagic flux. A previous study revealed that highly induced autophagy in epidermal cells affects their migratory capacity [17]. Furthermore, low SQSTM1 expression causes a decrease in autophagy levels, which in turn affects wound repair [34]. In the present study, autophagy flux was observed and monitored via western blot detection of MAPLC3B cleavage and mRFP–GFP–MAPLC3B fusion protein tracking of the formation and degradation of autophagosomes. We found that autophagosome production was significantly disrupted in epidermal cells treated with HG (35 mM) and autophagic flow was inhibited in a time-dependent manner, which was consistent with recent studies [17,34]. Furthermore, pharmacologic (BafA1) inhibition of autophagy worsened the already impaired epidermal cell function under HG conditions. We established an *in vivo* type 1 diabetes wound model and found significantly reduced autophagy in wounded epidermal cells compared with healthy control mice during wound repair. Based on the above evidence, autophagy appears to be a potential therapeutic target for refractory diabetic wounds and provides new insights into the pathogenesis of type 1 diabetes.

Based on the results of clinical trials, stem cell transplantation represents a potential treatment strategy for chronic diabetic wounds. ADSC-Exos are of great interest in cutaneous wound repair and regenerative medicine because of their unique properties. They can modulate the inflammatory response as well as promote wound cell proliferation, tissue regeneration and re-epithelialization and accelerate angiogenesis [21]. A previous study showed that ADSC-Exos regulate the Wnt/β-catenin pathway, which in turn affects keratinocyte migration and proliferation [35]. ADSC-Exos can promote wound repair in diabetic rats by increasing angiogenesis and optimizing fibroblast characteristics [36]. Although several studies have focused on the molecular biological mechanisms of ADSC-Exos during wound repair, their association with autophagy has been rarely reported [37,38]. Therefore, we examined the effect of ADSC-Exos on the regulation of diabetic trauma autophagy and the underlying molecular mechanisms. Our results indicated that ADSC-Exo therapy restores autophagy flux and cell function in epidermal cells damaged by HG treatment. Moreover, the beneficial effects of ADSC-Exos on epidermal cell function may be attenuated by inhibiting autophagy. Furthermore, we demonstrated that ADSC-Exos exhibit a positive effect on diabetic wound repair *in vivo*, primarily in the form of enhanced autophagy and accelerated epidermalization. Therefore, we propose that autophagy plays an important role in regulating ADSC-Exo-mediated diabetic wound repair; this finding may provide insights into the regulatory mechanism of ADSC-Exos in diabetic wound repair.

Finally, we investigated the mechanism of action of ADSC-Exos. We intersected DEGs with ARGs and found that *NAMPT*, *CD46*, *VAMP7*, *VAMP3* and *EIF2S1* may be key genes of ADSC-Exos that are involved in the specific regulation of cell autophagy in trauma healing. Interestingly, NAMPT is a rate-limiting enzyme in the Nicotinamide adenine dinucleotide (NAD) salvage pathway and is essential for NAD homeostasis. NAD is an important coenzyme in the process of energy production [39], and NAD depletion contributes to the death of autophagy/mitophagy-deficient cells [40]. NAMPT protects skin cells from ultraviolet A/B-mediated stress by restoring the transient depletion of NAD, thus promoting epidermal keratinocyte survival [41,42]. Based on these findings and our results, we hypothesized that ADSC-Exos may promote cell survival under hyperglycemic stress by activating the autophagy–NAMPT–NAD axis. However, the mechanism by which ADSC-Exos regulate traumatic autophagy remains unclear, and only some key molecules have been identified thus far, which need to be further examined.

## Conclusions

The present study provides a new approach for ADSC-Exos in the promotion of refractory wound repair. Our results revealed that the level of autophagy flux was down-regulated in the keratinocytes of the epidermis of patients with diabetes. Furthermore, ADSC-Exos were observed to upregulate autophagy flux, which resulted in increased epidermal cell proliferation and migration and promoted diabetic wound healing. Potential mechanisms through which ADSC-Exos exert their effects may involve the activation of the autophagy–NAD axis by increased *NAMPT* expression. However, the mechanism of action remains to be determined. We identified ADSC-Exo-based treatment as a potential pathway for diabetic wound repair and demonstrated its positive role in the regulation of cell autophagy for the first time. The findings of this study provide insights into the effect of ADSC-Exos on tissue repair and regenerative medicine and accelerate its clinical translation.

## Abbreviations

ACTB: Actin beta; ADSC-Exos: Adipose mesenchymal stem cell-derived exosomes; ARG: Autophagy-related genes; ANOVA: Analysis of variance; BafA1: Bafilomycin A1; CCK-8: Cell counting kit-8; DEG: Differentially expressed genes; DFU: Diabetic foot ulcer; EIF2S1: Eukaryotic translation initiation factor 2 subunit alpha; GO: Gene ontology; HaCaT: Human immortalized keratinocyte cell line; H&E: Hematoxylin and eosin; HG: High glucose; ICR: Institute of Cancer Research; KEGG: Kyoto encyclopedia of genes and genomes; MAP1LC3B: Microtubule associated protein 1 light chain 3 beta; NAMPT: Nicotinamide phosphoribosyltransferase; PBS: Phosphate-buffered saline; qRT-PCR: Quantitative reverse transcription polymerase chain reaction; ROS: Reactive oxygen species; SQSTM1: Sequestosome 1; TEM: Transmission electron microscopy; VAMP7: Vesicle-associated membrane protein 7.

## Supplementary Material

Figure_S1_tkae001

Supplementary_Material_tkae001

## Data Availability

Data are available upon request through the corresponding author.
