# Supplementary material for: Adipose mesenchymal stem cell-derived exosomes promote skin wound healing in diabetic mice by regulating epidermal autophagy
Source: Burns Trauma. 2024 Feb 29;12:tkae001. doi: 10.1093/burnst/tkae001 (PMC10905655; doi:10.1093/burnst/tkae001)
Supplement: Figure_S1_tkae001 [file figure_s1_tkae001.doc]

# Figure S1


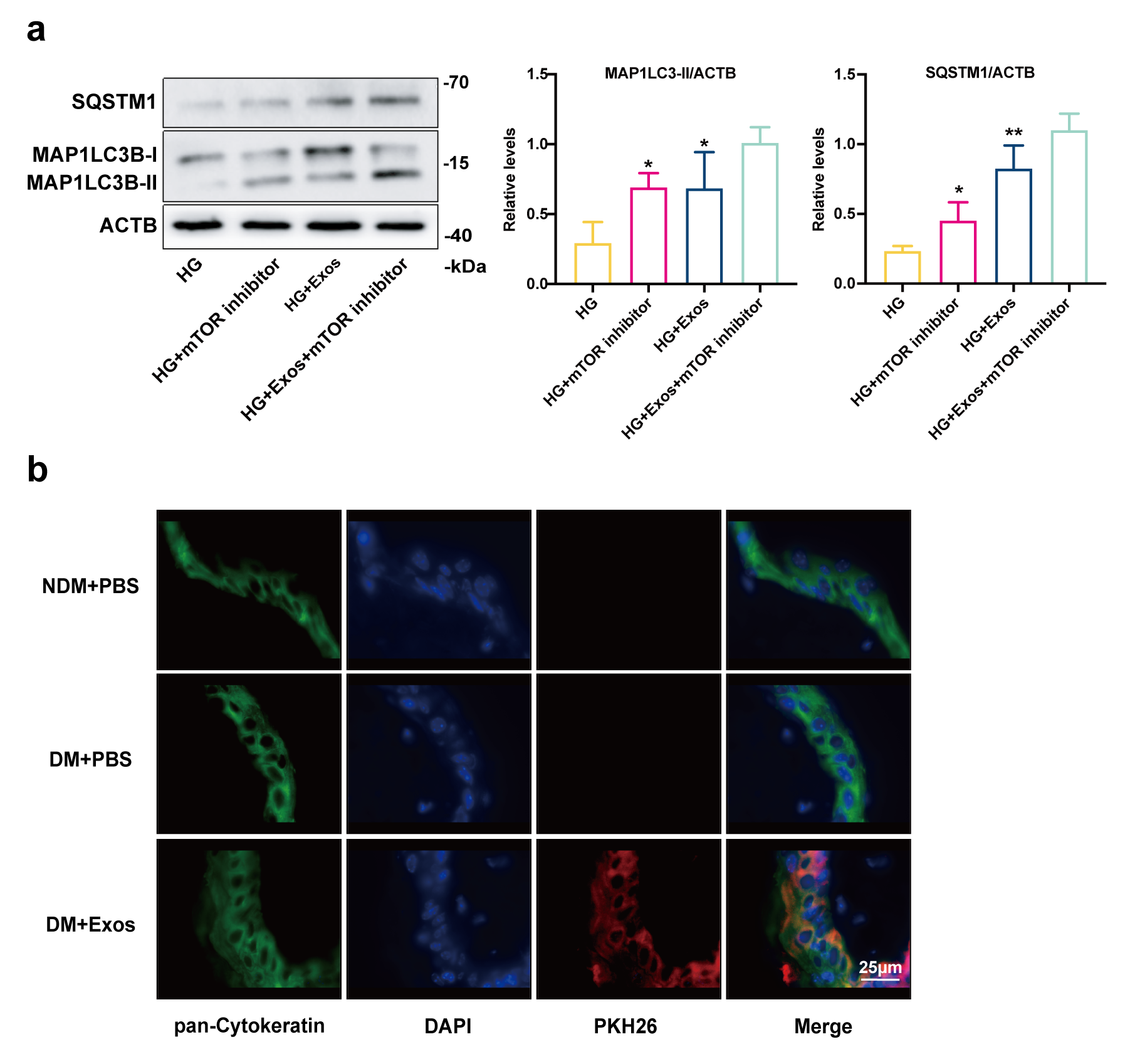


**Figure S1**. **HaCaT cells treated with an autophagy activator (mTORC1 inhibitor) as a positive control** **and exosome labelling and tracking assay** ***in vivo*** (**a**) Western blot analysis of MAP1LC3B-II and SQSTM1 expression levels in HaCaT cells treated with HG, HG + mTORC1 inhibitor, HG + ADSC-Exos (200 µg/ml) or HG + ADSC-Exos + mTORC1 inhibitor for 48 h. Quantitation of the MAP1LC3B-II/ACTB ratios are respectively shown (n = 3 independent experiments). (**b**) Fluorescent microscopy analysis of PKH26-labelled ADSC-Exo uptake by epidemic cells, scale bar: 25 μm. MAP1LC3B: microtubule associated protein 1 light chain 3 beta, SQSTM1: sequestosome 1, ACTB: actin beta, HaCaT: human immortalized keratinocyte cell line, mTORC1: mammalian target of rapamycin complex 1, ADSC-Exos: adipose mesenchymal stem cell-derived exosomes, NDM: non-diabetes mellitus mice, DM: diabetes mellitus mice, PBS: phosphate buffered saline.
